# Whole-cell (+)-ambrein production in the yeast *Pichia pastoris*

**DOI:** 10.1016/j.mec.2018.e00077

**Published:** 2018-08-16

**Authors:** Sandra Moser, Gernot A. Strohmeier, Erich Leitner, Thomas J. Plocek, Koenraad Vanhessche, Harald Pichler

**Affiliations:** a Austrian Centre of Industrial Biotechnology, Petersgasse 14, 8010 Graz, Austria; b Institute of Organic Chemistry, NAWI Graz, Stremayrgasse 9, 8010 Graz, Austria; c Institute of Analytical Chemistry and Food Chemistry, Graz University of Technology, NAWI Graz, Stremayrgasse 9, 8010 Graz, Austria; d ACS International S.A., 184 Route de St-Julien, CH-1228 Plan-les-Ouates, Switzerland; e Institute of Molecular Biotechnology, Graz University of Technology, NAWI Graz, BioTechMed Graz, Petersgasse 14/2, 8010 Graz, Austria

**Keywords:** *Aa*SHC, *Alicyclobacillus acidocaldarius* squalene-hopene cyclase, AOX1, alcohol oxidase, *Bme*TC, *Bacillus megaterium* terpene cyclase, BSM, basal salt medium, CDW, cell dry weight, FLD1, formaldehyde dehydrogenase 1, HRP, horse radish peroxidase, PTM1, Pichia trace metals, YNB, yeast nitrogen base, YPD, yeast extract peptone dextrose medium, Pichia pastoris, Metabolic engineering, Terpene cyclase, Triterpenoid, Squalene, (+)-ambrein

## Abstract

The triterpenoid (+)-ambrein is a natural precursor for (-)-ambrox, which constitutes one of the most sought-after fragrances and fixatives for the perfume industry. (+)-Ambrein is a major component of ambergris, an intestinal excretion of sperm whales that is found only serendipitously. Thus, the demand for (-)-ambrox is currently mainly met by chemical synthesis. A recent study described for the first time the applicability of an enzyme cascade consisting of two terpene cyclases, namely squalene-hopene cyclase from *Alicyclobacillus acidocaldarius* (*Aa*SHC D377C) and tetraprenyl-β-curcumene cyclase from *Bacillus megaterium* (*Bme*TC) for *in vitro* (+)-ambrein production starting from squalene. Yeasts, such as *Pichia pastoris,* are natural producers of squalene and have already been shown in the past to be excellent hosts for the biosynthesis of hydrophobic compounds such as terpenoids. By targeting a central enzyme in the sterol biosynthesis pathway, squalene epoxidase Erg1, intracellular squalene levels in *P. pastoris* could be strongly enhanced. Heterologous expression of *Aa*SHC D377C and *Bme*TC and, particularly, development of suitable methods to analyze all products of the engineered strain provided conclusive evidence of whole-cell (+)-ambrein production. Engineering of *Bme*TC led to a remarkable one-enzyme system that was by far superior to the cascade, thereby increasing (+)-ambrein levels approximately 7-fold in shake flask cultivation. Finally, upscaling to 5 L bioreactor yielded more than 100 mg L^−1^ of (+)-ambrein, demonstrating that metabolically engineered yeast *P. pastoris* represents a valuable, whole-cell system for high-level production of (+)-ambrein.

## Highlights

• Squalene accumulation in *P. pastoris* for triterpenoid biosynthesis.
• First whole-cell biosynthesis of (+)-ambrein in yeast.
• Cell and enzyme engineering improved (+)-ambrein yield to> 100 mg L^−1^ culture.

## Graphical abstract

**Figure:**
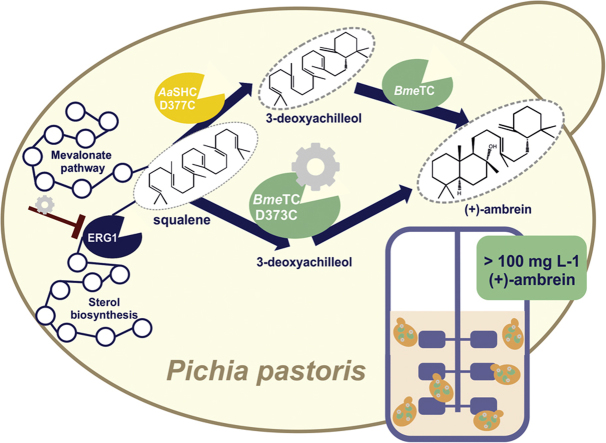

## 1 Introduction

**Fig. 1:**
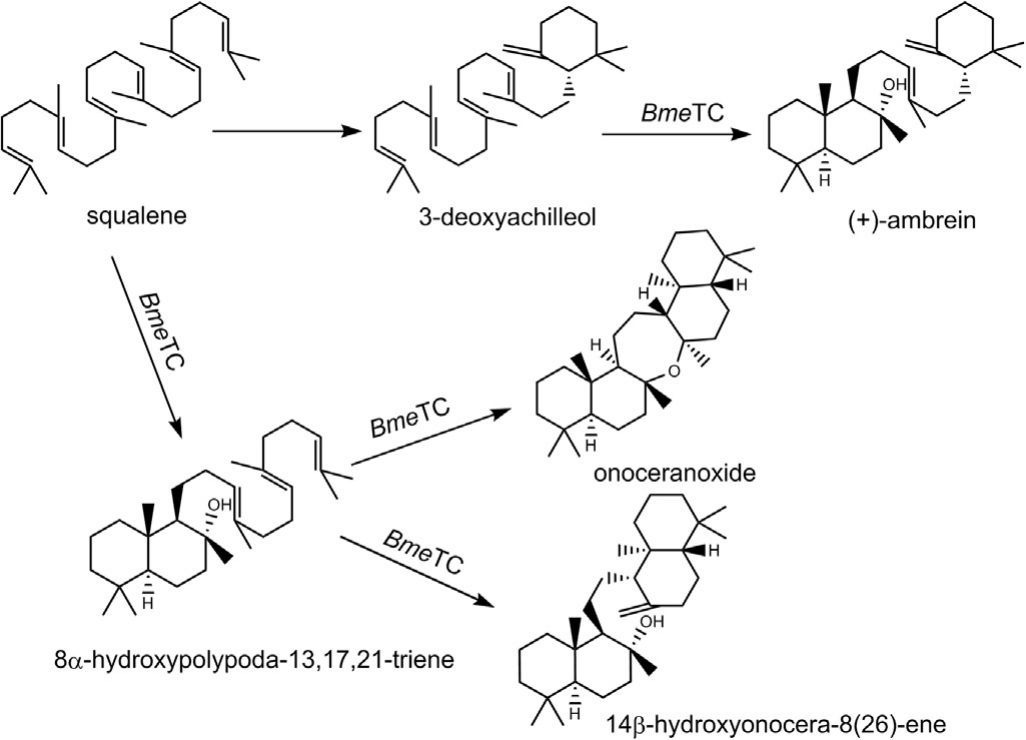
Generation of (+)-ambrein from squalene using *Aa*SHC D377C and *Bme*TC according to Ueda et al. (2013).

The triterpenoid (+)-ambrein is a major component of ambergris, an intestinal excretion of the sperm whale that represents one of the most valuable animal resources for perfume production. Apart from (+)-ambrein, ambergris also contains several cholestenol-type sterols (Ohloff, 1982). Upon exposure to sea water, sun light and air, (+)-ambrein undergoes oxidative degradation, yielding (-)-ambrox and several other odor-active compounds (Sell, 2006). This natural process can be simulated by reacting pure (+)-ambrein with singlet oxygen, thereby yielding several photo-oxidation products, including ambrox, γ-coronal, α-ambrinol and dehydroambraoxid (Ohloff, 1990). Beyond application in the perfume industry, animal studies have also demonstrated the potential anti-nociceptive (Taha, 1992) and aphrodisiac (Taha et al., 1995) properties of (+)-ambrein as well as possible effects on the cardiovascular system (Raza et al., 1999) and on smooth muscle response (Taha et al., 1998). As the natural ambergris supply is highly limited, total or partial syntheses (reviewed by Zerbe and Bohlmann, 2015) have been developed for production of (+)-ambrein, (-)-ambrox and related compounds. A recent study by Ueda et al. (2013) described the possibility to produce (+)-ambrein from squalene applying only two enzymes (Fig. 1). Squalene-hopene cyclase variant D377C from *Alicyclobacillus acidocaldarius* (*Aa*SHC D377C) produces 3-deoxyachilleol (Sato and Hoshino, 1999), which can be converted to (+)-ambrein by a second enzyme, a versatile tetraprenyl-β-curcumene cyclase from *Bacillus megaterium* (*Bme*TC) first described by Sato et al. (2011).

For the generation of (+)-ambrein, the authors incubated *Escherichia coli* cell-free extracts expressing the aforementioned terpene cyclases with squalene or 3-deoxyachilleol, respectively. The latter had been purified in between of the two conversion steps. This, and the relatively low yield rendered the described approach not immediately feasible for industrial approaches (Ueda et al., 2013). Moreover, employing squalene as a substrate significantly increases process costs. In contrast to *E. coli*, yeasts naturally produce triterpenoid precursors, such as squalene or 2,3-oxidosqualene *via* their intrinsic mevalonate and sterol biosynthesis pathway. Furthermore, yeasts can easily be genetically manipulated and, for these reasons, represent ideal hosts for terpenoid production as reviewed, for example, by Wriessnegger and Pichler (2013) or Leavell et al. (2016). Although most studies addressing terpenoid biosynthesis in yeast focus on sesquiterpenoids (C_15_) or carotenoids (C_40_), a few have also been successful in establishing yeast, especially *Saccharomyces cerevisiae*, as a production platform for triterpenoids (C_30_). This concerns mainly ginsenosides, through metabolic engineering (Dai et al., 2013, Kirby et al., 2008, Madsen et al., 2011), or cell engineering approaches (Arendt et al., 2017) and modified cultivation procedures (Moses et al., 2013). Though not yet as well-studied as *S. cerevisiae*, where metabolic engineering of the mevalonate pathway for terpenoid precursor production is well-described (Ro et al., 2006), the methylotrophic yeast *Pichia pastoris* exhibits some properties that render it highly interesting as a production platform. The success of recombinant membrane protein expression in *P. pastoris* has been shown numerous times (reviewed by Byrne, 2015; Emmerstorfer et al., 2014). Its ability to grow to very high cell densities, *i.e.*> 100 g/L cell dry weight in bioreactors, makes it very attractive for industrial purposes (Cereghino et al., 2002). Furthermore, its applicability for terpenoid production has been demonstrated in several studies (Liu et al., 2015, Wriessnegger et al., 2016, Wriessnegger et al., 2014, Zhao et al., 2016). To our knowledge, the work by Liu et al. (2015) is the only one to date that describes metabolic engineering of *P. pastoris* for heterologous triterpenoid production. In brief, expression of *ERG1* (squalene epoxidase) was increased while *ERG7* (lanosterol synthase) expression, the next protein in ergosterol biosynthesis pathway, was downregulated to accumulate 2,3-oxidosqualene, the precursor for dammarenediol-II. Furthermore, cultures were supplemented with squalene, which significantly enhanced productivity. In contrast to this approach, to generate sufficient amounts of squalene for (+)-ambrein synthesis, our strategy aimed at downregulating *ERG1* expression and activity. In *S. cerevisiae*, Erg1 activity had been successfully decreased in several studies utilizing the inhibitor terbinafine, which resulted in clearly enhanced levels of squalene (Garaiová et al., 2014, Han et al., 2018, Klobučníková et al., 2003), while this study represents the first analysis of the effects of terbinafine on Erg1p in *P. pastoris*. In addition to supplementing terbinafine, also the possibility to downregulate expression of *ERG1* was evaluated. Therefore, the native promoter of *ERG1* was exchanged for the regulatable *PIS1* promoter, which can be partially repressed using zinc or inositol (Delic et al., 2013). On top of converting 3-deoxyachilleol to (+)-ambrein, *Bme*TC can convert squalene to 8α-hydroxypolypoda-13,17,21-triene and, subsequently, 14β-hydroxyonocera-8(26)-ene and onoceranoxide (Fig. 1) (Ueda et al., 2013). To ensure that sufficient amounts of 3-deoxyachilleol can be formed from squalene by *Aa*SHC D377C while reducing the formation of 8α-hydroxypolypoda-13,17,21-triene, a sequential cultivation and expression strategy was developed. The first phase was dedicated to cell growth and squalene accumulation. At the beginning of the second phase, expression of *Aa*SHC D377C controlled by the *FLD1* promoter (Shen et al., 1998) was induced with methylamine. During the third and last phase of cultivation, both *Aa*SHC D377C and *Bme*TC were expressed employing the *AOX1* promoter (Tschopp et al., 1987) and using methanol (MeOH) as inducer. Another essential part of this study was to develop analytical methods that allowed us to detect and quantify the different triterpenoids extracted from engineered *P. pastoris* strains as the GC-MS method described by Ueda et al. (2013) cannot be used to separate the highly similar compounds such as squalene and 3-deoxyachilleol, or 8α-hydroxypolypoda-13,17,21-triene and (+)-ambrein. Following these approaches, together with engineering of *Bme*TC towards a bifunctional enzyme, we managed to establish *P. pastoris* as the first eukaryotic host for whole-cell production of (+)-ambrein with yields that render it highly interesting for future industrial applications.

## 2 Material&methods

### 2.1 Vector and strain construction

*E. coli* TOP10F′ (F′[*lac*I^q^ Tn10(tet^R^)] *mcr*A Δ(*mrr-hsd*RMS-*mcr*BC) φ80*lac*ZΔM15 Δ*lac*X74 *nup*G *rec*A1 *ara*D139 Δ(*ara-leu*)7697 *gal*U *gal*K *rps*L(Str^R^) *end*A1 λ^−^) from life technologies, Vienna, Austria was used for vector construction and amplification. *P. pastoris* strains constructed in this study were based on strains CBS7435 *his4* and CBS7435 *his4 ku70* (Näätsaari et al., 2012). Plasmid backbones employed for strain constructions in *P. pastoris* had been described in the same work. All strains described in this work are listed in Table 1.

**Table 1:** Strains used in this study.

**Strain**	**Description**	**Source**
**Wildtype (WT)**	CBS7435 *his4*	Näätsaari et al. (2012)
**WT ku70**	CBS7435 *his4 ku70*	Näätsaari et al. (2012)
**P**_**PIS1**_**-*****ERG1***	CBS7435 *his4,* p*Pp*HIS4[P_PIS1_-*ERG1*]	This study
**WT ku70 SHC**	CBS7435 *his4 ku70,* p*Pp*Kan[*Aa*SHC D377C]	This study
**WT TC**	CBS7435 *his4,* p*Pp*FZE[*Bme*TC]	
**P**_**PIS1**_**-*****ERG1*****SHC**	CBS7435 *his4,* p*Pp*HIS4[P_PIS1_-*ERG1*], p*Pp*Kan[*Aa*SHC D377C],	This study
**P**_**PIS1**_**-*****ERG1*****TC**	CBS7435 *his4,* p*Pp*HIS4[P_PIS1_-*ERG1*], p*Pp*FZE[*Bme*TC]	
**P**_**PIS1**_**-*****ERG1*****SHC TC**	CBS7435 *his4,* p*Pp*HIS4[P_PIS1_-*ERG1*], p*Pp*Kan[*Aa*SHC D377C], p*Pp*FZE[*Bme*TC]	This study
**P**_**PIS1**_**-*****ERG1*****TC D373C**	CBS7435 *his4,* p*Pp*Kan[*Bme*TC D373C]	This study

**Fig. S1:**
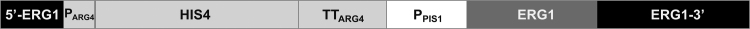
Expression cassette to exchange the native promoter of ERG1 by the PIS1 promoter. Expression cassette contains homologous regions to the 5′- and 3′-flanking sequences of the ERG1 locus.

**Fig. S2:**
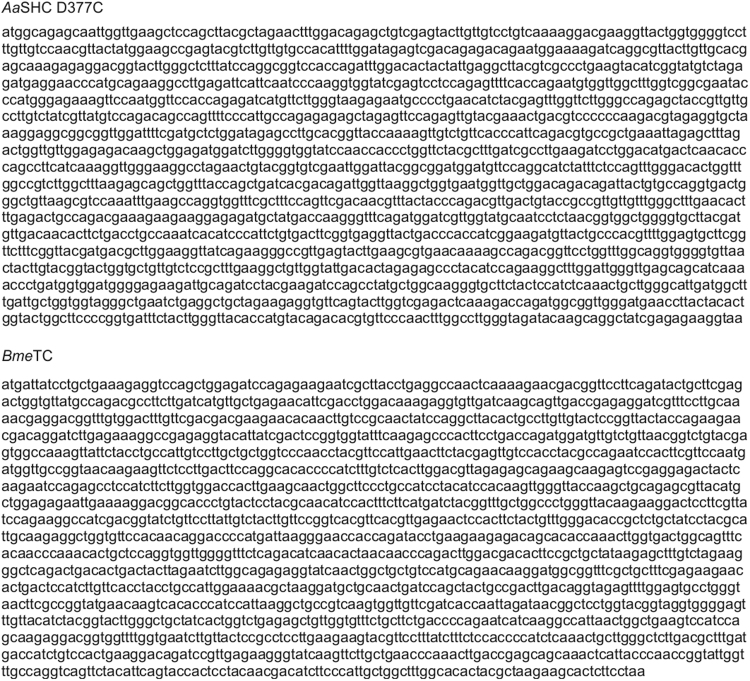
AaSHC and BmeTC sequences codon-optimize for expression in P. pastoris.

**Fig. S3:**
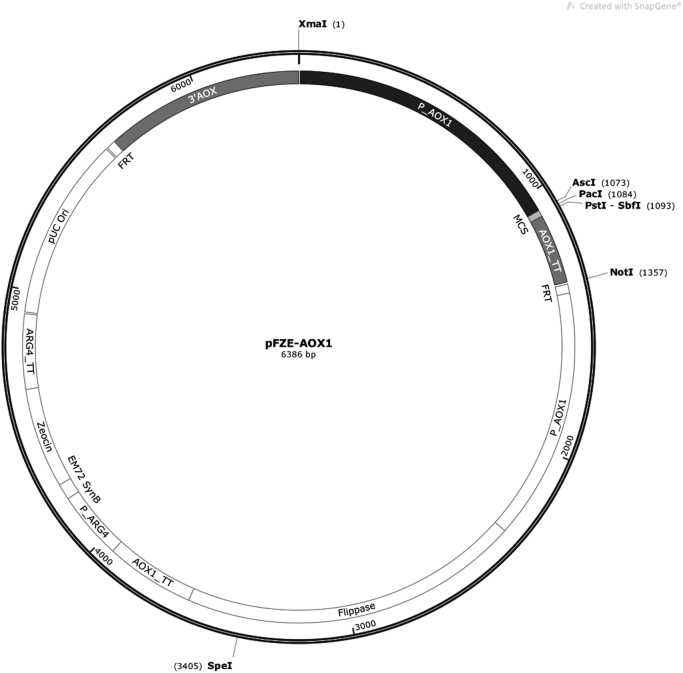
Map of plasmid pFZE-AOX1.

Phusion^®^ High Fidelity DNA polymerase (Thermo Fisher Scientific Inc., St. Leon-Rot, Germany) was used for gene amplification according to the recommended PCR protocol (for primer sequences see Table S1). To exchange the native promoter of *ERG1* for the *PIS1* promoter, an integrative expression plasmid containing the following elements was assembled: *PIS1* promoter (primers 5&6), *ERG1* coding sequence (GenBank number: LT962478.1, bases 1999855–2001333), 5′ (primers 1&2) and 3′ (including *ERG1* gene; primers 7&8) untranslated regions of the *ERG1* locus for homologous integration were all amplified from genomic DNA of strain CBS7435 *his4. HIS4* selection marker (primers 3&4) was amplified from p*Pp*HIS4 and origin of replication and kanamycin resistance cassette (primers 13&14) were amplified from pET-28. These parts including overlapping sequences were amplified by PCR and were joined by Gibson assembly. Prior to *P. pastoris* transformation, the plasmid was digested with selected restriction enzymes (Thermo Scientific, St. Leon-Rot, Germany) to generate an integration cassette flanked by homologous sequences for targeted integration into the *ERG1* locus (Fig. S1). Correct integration was confirmed through colony PCR (primer pairs 9&10 and 11&12). *Aa*SHC D377C (GenBank number of native gene: AB007002.1) and *Bme*TC (GenBank number of native gene: CP001982.1, 2130781–2132658) were manually codon-harmonized for expression in *P. pastoris.* Therefore, the frequency of each codon occurring in the respective gene was analyzed for *P. pastoris* as well as for the originating organism (*A. acidocaldarius* or *B. megaterium*) using the gcua tool (http://gcua.schoedl.de/) (Fuhrmann et al., 2004) in combination with codon usage tables provided by http://www.kazusa.or.jp (Nakamura et al., 2000). For frequently used codons in the originating organism, frequently occurring codons in *P. pastoris* were chosen while codons with medium and low frequency were substituted with ones that occur with medium frequency in *P. pastoris*. These synthetic genes were purchased from GeneArt^®^ (Fig. S2). For expression of *Aa*SHC D377C, the *AOX1* promoter of the p*Pp*Kan expression vector was exchanged for the *FLD1* promoter (primers 15&16; amplified from CBS7435 *his4* genome) by subcloning with *Swa*I and *Spe*I. The synthetic gene encoding *Aa*SHC D377C was amplified (primers 17&18) to encode an N-terminal FLAG-tag sequence and to add *Spe*I and *Not*I restriction sites for integration into the expression vector as well as a Kozak sequence (CGAAACG). For expression of *Bme*TC, a novel expression vector named pFZE (Fig. S3) was designed by amplifying *AOX1* promoter (primers 23&24) and terminator (primers 25&26) from p*Pp*T4α, 3′*AOX1* sequence (primers 29&30) from pAaHSwa (Ahmad et al., 2014) and the flippase cassette with Zeocin resistance (primers 27&28) from p*Pp*KC1 (Mudassar Ahmad, manuscript in preparation). Subsequently, all parts were assembled through Gibson cloning. The synthetic *Bme*TC gene was amplified (primers 19&20) to add *Asc*I and *Pac*I sites as well as Kozak sequence and to encode an N-terminal *myc*-tag sequence. To generate and express *Bme*TC D373C variant, the *Bme*TC sequence including Kozak sequence and *myc*-tag was first amplified from p*Pp*FZE[*Bme*TC] with primers 21&22 containing *Spe*I and *Not*I restriction sites for subcloning into p*Pp*Kan_pFLD. For amino acid exchange D373C, a slightly modified protocol of Stratagene's QuikChange site-directed mutagenesis kit was applied using Phusion^®^ High Fidelity DNA polymerase. In brief, 25 µL of two separate mutagenesis PCR mixtures were prepared, that contained either the forward or the reverse primer (primers 31&32). After five cycling steps performed according to the Stratagene manual, the two PCR reactions were combined and PCR was continued for another 13 cycles (Edelheit et al., 2009). In general, all cloned and modified sequences were checked by sequencing (Microsynth AG, Balgach, Switzerland). Expression vectors were linearized with *Smi*I (p*Pp*Kan_pFLD) or *Sma*I (p*Pp*FZE) and were transformed into electrocompetent *P. pastoris* cells according to the protocol of Lin-Cereghino et al. (2005). Aliquots were plated on histidin-free minimal media or on YPD plates containing either 25 mg L^−1^ Zeocin™ (InvivoGen, Vienna, Austria) or 300 mg L^−1^ geneticin sulfate (Formedium™, Norfolk, United Kingdom), respectively.

### 2.2 Media and strain cultivation

*P. pastoris* cultures were grown in YPD containing 1% yeast extract, 2% peptone (both obtained from Becton, Dickinson and Company, Schwechat, Austria) and 2% glucose (Carl Roth GmbH&Co. KG, Karlsruhe, Germany). Minimal dextrose (MD) plates (1.34% Difco™ yeast nitrogen base w/o amino acids (YNB, from Becton, Dickinson and Company, Schwechat, Austria), 4 × 10^−5^% biotin, 2% dextrose) were used for selection of strains containing the p*Pp*HIS4 expression vector. *E. coli* was cultivated in LB medium (Lennox) purchased from Carl Roth GmbH&Co. KG, Karlsruhe, Germany. Media for plates were solidified by addition of agar to 1.5%. Pre-cultures were grown in YPD medium at 28 °C and 130 rpm overnight. Main cultures of 50 mL YPD in baffled 300 mL shake flasks covered with cotton cloth were inoculated to an OD_600_ of 0.1 and were cultivated at 28 °C and 130 rpm. After 24 h of growth, for induction of P_FLD1_ but not of P_AOX1_, 12.5 µL of a 40% methylamine solution in H_2_O (Sigma-Aldrich^®^, Vienna, Austria) were added to the culture. Alternatively, to induce P_FLD1_ and/or P_AOX1_, methanol was added to a final concentration of 0.75% every 12 h. To increase intracellular squalene accumulation, 5 µL of 1 mg L^−1^ terbinafine hydrochloride (Sigma-Aldrich^®^, Vienna, Austria) solution in ethanol was added to the cultures in the beginning and every 48 h of cultivation.

### 2.3 Expression analysis

Sample preparation for SDS-PAGE was carried out according to the method of Riezman et al. (1983), with slight modifications. In brief, 3 OD_600_ units taken after 48 h of induction were transferred to 1.5 mL reaction tubes, were centrifuged at 2000 rpm for 5 min and the supernatants were removed. Cell pellets were resuspended in 300 µL of 1.85 M NaOH (7.5% β-mercaptoethanol) and were incubated on ice for 10 min. Then, 300 µL of 50% (w/v) trichloroacetic acid were added and following incubation on ice for 1 h, the samples were centrifuged at maximum speed for 5 min at 4 °C. After removing the supernatants, the cell pellets were washed with 500 µL of ddH_2_O, resuspended in 50 µL of loading dye (NuPAGE®) and were incubated for 10 min at 70 °C. Cell debris was spun out and 15 µL aliquots were loaded onto an SDS-PAGE gel. Protein levels were checked by immunoblotting using primary antibodies against FLAG- or c-*myc*-tags (F1804 and C3956 from Sigma-Aldrich^®^, Vienna, Austria). HRP-conjugated secondary antibodies (A4416 and A9169 from Sigma-Aldrich^®^, Vienna, Austria) and enhanced chemiluminescent signal detection (SuperSignal™, Pierce Biotechnology, Rockford, IL) were used to visualize immunoreactive bands. SDS–PAGE and immunoblotting were performed according to the manual of the NuPAGE® SDS–PAGE Gel System (life technologies, Vienna, Austria). For determination of intracellular localization of heterologous proteins, cells were harvested after 24 h of induction and cell lysis and fractionation was performed as described by Geier et al. (2012). Fifteen µg of protein of total cell lysate, cytosolic fraction and microsomal fraction was precipitated with trichloroacetic acid and was subsequently loaded onto SDS-PAGE. Protein expression levels were analyzed by immunoblotting as described above.

### 2.4 Quantification of triterpenoids and sterols by GC-MS and GC-FID

Sterol extraction of cell culture volumes corresponding to 10 OD_600_ units was performed essentially as described by Hirz et al. (2013). In brief, cells were resuspended in 0.6 mL of methanol, 0.4 mL of 0.5% pyrogallol in methanol, and 0.4 mL of 60% KOH. Five µL of a 2 mg mL^−1^ cholesterol solution in ethanol were added as internal standard. After heating the samples for 2 h at 90 °C, saponified lipids were extracted two times with 1 mL of *n*-heptane. Dried extracts were dissolved in 10 µL of pyridine and were derivatized with 50 µL of *N,O*-bis(trimethylsilyl)trifluoroacetamide. Samples were diluted with 200 µL of ethyl acetate and analyzed by gas chromatography–mass spectrometry (GC-MS) or gas chromatography - flame ionization detector (GC-FID). Quantification of analytes was performed by correlating the peak area of the internal standard cholesterol to the peak area of the respective compound.

#### 2.4.1 GC-MS method

A 7.5 m OPTIMA® delta-6 column (Macherey-Nagel; polysiloxane phase with autoselectivity 0.10 mm × 0.10 µm) was used on a Shimadzu QP2010 plus GCMS system equipped with a single quadrupole mass filter with electron impact ionization (EI 70 eV). Sample aliquots of 1 µL were injected in split mode (split ratio 15:1) at 270 °C injector and 300 °C detector temperatures with hydrogen as carrier gas at constant flow rate of 60 cm s^−1^. The oven temperature program was as follows: 70 °C for 1 min, 30 °C min^−1^ ramp to 320 °C (3 min). MSD was operated in a mass range of 50–550 amu with 6.6 scans/s and at electron multiplier voltage of 1.10 kV.

#### 2.4.2 GC-FID method

For routine analysis, a GC-FID method was developed. Therefore, a OPTIMA® delta-6 column (Macherey-Nagel; polysiloxane phase with autoselectivity; 7.5 m × 0.10 mm×0.10 µm) on a Hewlett-Packard 6890 GC equipped with a flame ionization detector (FID) was used. Sample aliquots of 1 µL were injected in split mode (split ratio 30:1) at 250 °C injector temperature and 320 °C detector temperature with hydrogen as carrier gas and a flow rate set to 0.4 mL min^−1^ in constant flow mode (58 cm s^−1^ linear velocity). The oven temperature program was as follows: 70 °C for 1 min, 30 °C min^−1^ ramp to 310 °C (1 min).

### 2.5 Purification and NMR analysis of 3-deoxyachilleol

An ethyl acetate extract (40 mL) of metabolites from cell lysate - obtained from 700 mL of WT ku70 SHC fermentation broth using a Merckenschlager (MSK) homogenizer (Sartorius, Goettingen, Germany) as described by Hirz et al. (2013) - was concentrated under reduced pressure and the residue purified *via* flash chromatography on silica gel (0.035–0.070 mm, 60 Å, Acros Organics) using cyclohexane as eluent. All fractions containing a pure compound at an R_f_ value of 0.39 (cyclohexane) were pooled and concentrated under reduced pressure. The isolated amount was 6.0 mg and the material confirmed as 3-deoxyachilleol by GC-MS and NMR analysis.

### 2.6 Bioreactor cultivation of strain P_PIS1_-ERG1 TC D373C

For bioreactor cultivation, “*Pichia* Fermentation Process Guidelines” (Invitrogen) were followed, with some minor adjustments. Precultures were grown in 300 mL baffled shake flasks containing 50 mL of buffered complex glycerol medium, BMGY, composed of 1% yeast extract, 2% peptone, 100 mM potassium phosphate, pH 6.0, 1.34% YNB, 4 × 10^−5^% biotin and 1% glycerol, at 28 °C and 130 rpm for 40 h. Seed culture in 2 L baffled shake flasks containing 300 mL of BMGY were inoculated to an OD_600_ of 0.3 and were grown for 24 h at 28 °C and 110 rpm. For bioreactor cultivation, BIOSTAT® CT+ bioreactor system (Sartorius BBI Systems GmbH, Melsungen, Germany) was used. Batch cultivation was performed in defined basal salt medium (BSM, per liter 0.17 g CaSO_4_·2H_2_O, 2.86 g K_2_SO_4_, 0.64 g KOH, 14 g MgSO_4_·7H_2_O, 4.25 g H_3_PO_4_, 0.22 g NaCl, 40 g glycerol, 12 mL PTM1 (per liter 5.0 mL of H_2_SO_4_ (69%), 5.99 g CuSO_4_·5H_2_O, 1.18 g KI, 3.0 g MnSO_4_·H_2_O, 0.2 g Na_2_MoO_4_·2H_2_O, 0.02 g H_3_BO_3_, 0.92 g CoCl_2_·6H_2_O, 42.18 g ZnSO_4_·7H_2_O and 65.0 g FeSO_4_·7H_2_O) and4.35 mL of 0.02% of biotin). At the start of the batch phase, 3.5 L of BSM (containing 0.1 µg mL^−1^ terbinafine) were inoculated to an OD_600_ of 1. Dissolved oxygen (dO_2_) was monitored with a dO_2_ electrode. The inlet-gas flow rate was set to 3 L/min and agitation rate was adjusted automatically to keep dissolved oxygen levels> 30%. The pH was measured with an autoclavable pH-electrode and controlled at pH 5.0 by automatic addition of 25% NH_3_. Batch culture was grown until glycerol was completely consumed, which was indicated by dO_2_ increase (dO_2_ spike). Then, glycerol fed-batch was initiated by exponential addition of glycerol solution (50% w/v containing 12 mL L^−1^ of PTM1 and 4.35 mL of 0.02% of biotin). Feeding was continued over 19 h with a feed rate of 50 g h^−1^. After the complete consumption of glycerol, which was indicated by a dO_2_ spike, methanol feeding was started at a feeding rate of 17.5 g h^−1^ and continued for 74 h. After 24 h of induction, 0.2 mg L^−1^ of terbinafine were added. Biomass concentration in cultivation broth was determined gravimetrically as cell dry weight (CDW). One mL samples of the cell culture were centrifuged in pre-weighed 1.5 mL reaction tubes for 5 min at 13.200 rpm. The supernatants were removed and the pellets were dried at 100 °C in an oven for two days.

## 3 Results and discussion

In order to generate a *P. pastoris* strain capable of producing (+)-ambrein, several engineering approaches were employed. First, intracellular levels of the precursor of (+)-ambrein, squalene, were significantly enhanced by reducing its flux towards ergosterol biosynthesis. Secondly, the enzyme cascade consisting of *Aa*SHC D377C and *Bme*TC was heterologously expressed. To analyze all products resulting from the activity of these two enzymes in whole cells, analytical methods including GC-FID and GC-MS were developed and refined that enabled separation of several of these highly similar compounds. Additionally, engineering of *Bme*TC (D373C) led to a novel, more versatile enzyme that is able to catalyze the conversion starting from squalene to 3-deoxyachilleol and further to (+)-ambrein far more efficiently than the previously described two-enzyme cascade. Finally, the potential of the resulting *P. pastoris* strain for (+)-ambrein production at larger scale was demonstrated in a 5 L bioreactor cultivation.

### 3.1 Increasing squalene levels by targeting ERG1 expression and activity

**Fig. 2:**
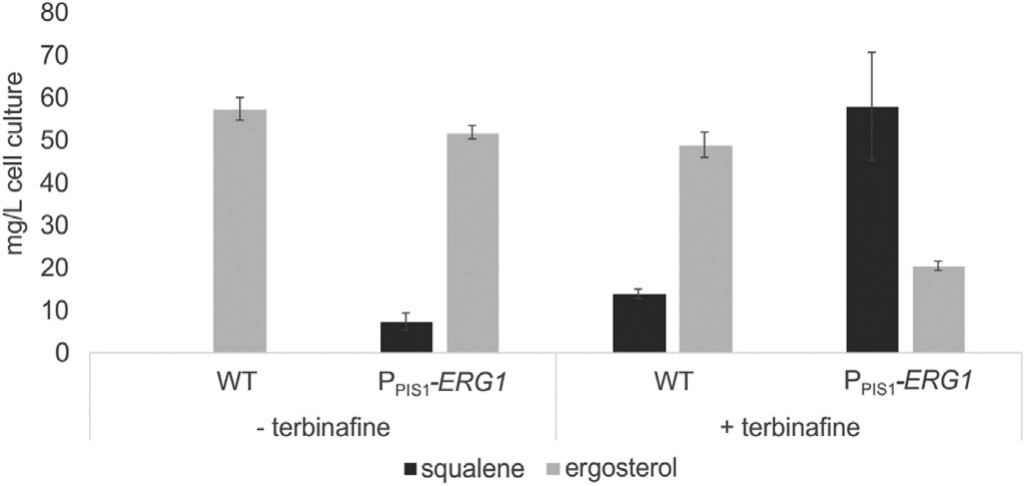
Intracellular squalene accumulation in strains WT and P_PIS1_-*ERG1* after cultivation for 24 h in YPD, without or with 0.1 µg mL^−1^ of terbinafine. Mean values and standard deviations of biological triplicates are given.

Under standard cultivation conditions, squalene levels in wild type *P. pastoris* are usually below detection limit (Adelantado et al., 2017). To our knowledge, the only study on triterpenoid production in *P. pastoris* in which proteins involved in sterol biosynthesis were targeted focused on accumulation of 2,3-oxidosqualene (Liu et al., 2015). To increase intracellular squalene supply for (+)-ambrein biosynthesis, two strategies, both targeting Erg1p activity, were employed. First, the applicability of the Erg1p inhibitor terbinafine that has been described before to be beneficial for squalene accumulation in *S. cerevisiae* (Garaiová et al., 2014) was tested on *P. pastoris*. Initial tests revealed that concentrations of up to 6 µg mL^−1^ of terbinafine did not impair growth of *S. cerevisiae,* while *P. pastoris* growth was clearly reduced within the first 24 h of cultivation at terbinafine levels higher than 0.1 µg mL^−1^ due to a prolonged lag-phase. Compared to the values obtained with 0.1 µg mL^−1^, a concentration of 0.2 µg mL^−1^ of terbinafine resulted in 50% reduced optical density while supplementation with 0.4 µg mL^−1^ terbinafine reduced growth of *P. pastoris* by approximately 80% after 24 h of cultivation. Therefore, the concentration of 0.1 µg mL^−1^ of terbinafine was used for all experiments. Quantification of squalene and ergosterol levels upon cultivation with or without terbinafine showed a strong effect of terbinafine on squalene levels, yielding 14 mg L^−1^ after 24 h of cultivation (Fig. 2). Additionally, the native promoter of *ERG1* was replaced by the *PIS1* promoter that can be regulated through zinc and inositol levels in cultivation medium (Delic et al., 2013). Significant levels of squalene could be detected in strain P_PIS1_-*ERG1* after 24 h of cultivation, although slightly lower than the amount obtained with terbinafine supplementation. Interestingly, these two approaches could be combined and yielded 58 mg L^−1^ of squalene, which is markedly more than just the effects of each of the two approaches added up (Fig. 2). Furthermore, for the combined approach an effect on sterol biosynthesis was visible as ergosterol levels were clearly decreased.

Apparently, the amounts of zinc or inositol present in yeast extract are suitable to achieve partial repression of the *PIS1* promoter while not completely inhibiting sterol biosynthesis, which would likely impair viability and growth of the cells. These experiments also showed that after 48 h of cultivation, the growth-impairing effect of terbinafine (concentrations up to 0.4 µg mL^−1^) was diminished and all cultures reached the same optical density at this time point irrespective of the tested terbinafine concentration. This might either be due to a possible instability of terbinafine at 28 °C over time or to the possibility that, despite a partial inhibition of *ERG1*, 48 h constituted a sufficient time range to synthesize the amount of sterols essential for cell growth. Thus, this time point was chosen to repeat supplementation with terbinafine during longer cultivations. To our knowledge, this is the first time that accumulation of high levels of squalene in *P. pastoris* was achieved.

### 3.2 Expression of AaSHC D377C and BmeTC in *P. pastoris*

**Fig. 3:**
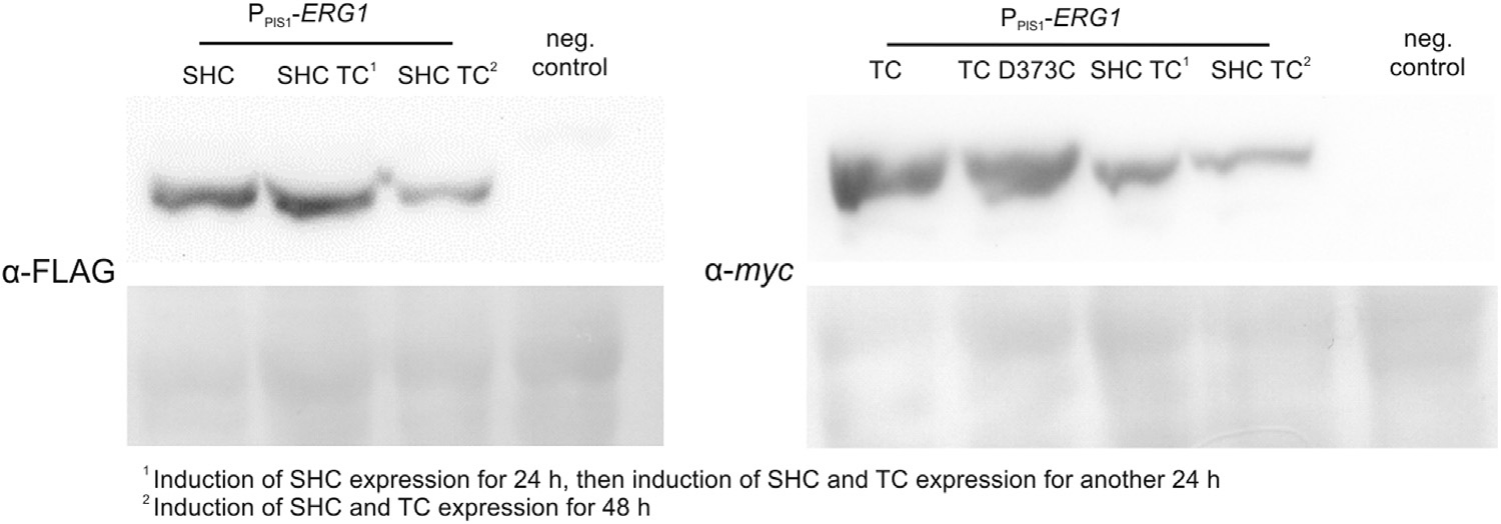
Immunoblot analysis using antibodies directed against FLAG-tag (FLAG-*Aa*SHC D377C) and *myc*-tag (*myc*-TC and *myc*-TC D373C). P_PIS1_-*ERG1* TC was employed as negative control for α-FLAG detection while P_PIS1_-*ERG1* SHC D377C was used as negative control for α-myc detection. PonceauS stain of the nitrocellulose membranes was performed as control of transferred protein amount.

The first step towards whole-cell (+)-ambrein production was to test if the two terpene cyclases described by Ueda et al. (2013), *Aa*SHC D377C and *Bme*TC, could be expressed in yeast. Therefore, codon-harmonized genes were designed and protein levels after 48 h of induction of the respective protein were assessed by immunoblotting (Fig. 3). Both proteins could successfully be expressed in *P. pastoris*. While there was hardly any difference in *Aa*SHC D377C amounts between strain P_PIS1_-*ERG1* SHC D377C and P_PIS1_-*ERG1* SHC D377C TC, expression levels of *Bme*TC were clearly lower in the strain co-expressing *Aa*SHC D377C when the sequential expression strategy was employed.

**Fig. S4:**
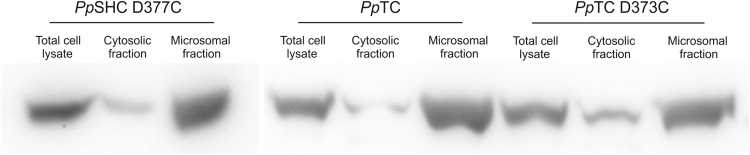
Western Blot analysis using antibodies directed against FLAG-tag of FLAG-SHC D377C and myc-tag of myc-TC and myc-TC D373C in the respective P. pastoris (Pp) expression strains.

Localization studies (Fig. S4) revealed that both proteins were primarily found to be membrane-associated, which correlated well with the findings of Seckler and Poralla (1986) determining *Aa*SHC to be membrane-associated in *A. acidocaldarius*. With *Aa*SHC D377C being expressed about 24 h earlier than TC, membrane space to accommodate *Bme*TC might be limited. This hypothesis was supported by immunoblot analysis (Fig. 3) of P_PIS1_-*ERG1* SHC D377C TC samples from cultivations during which expression of *Aa*SHC D377C and *Bme*TC was induced simultaneously using MeOH. In this case, expression levels of both proteins were clearly lower compared to the strains expressing only one of the two enzymes.

### 3.3 Analysis of AaSHC D377C and BmeTC products in whole cells

**Fig. 4:**
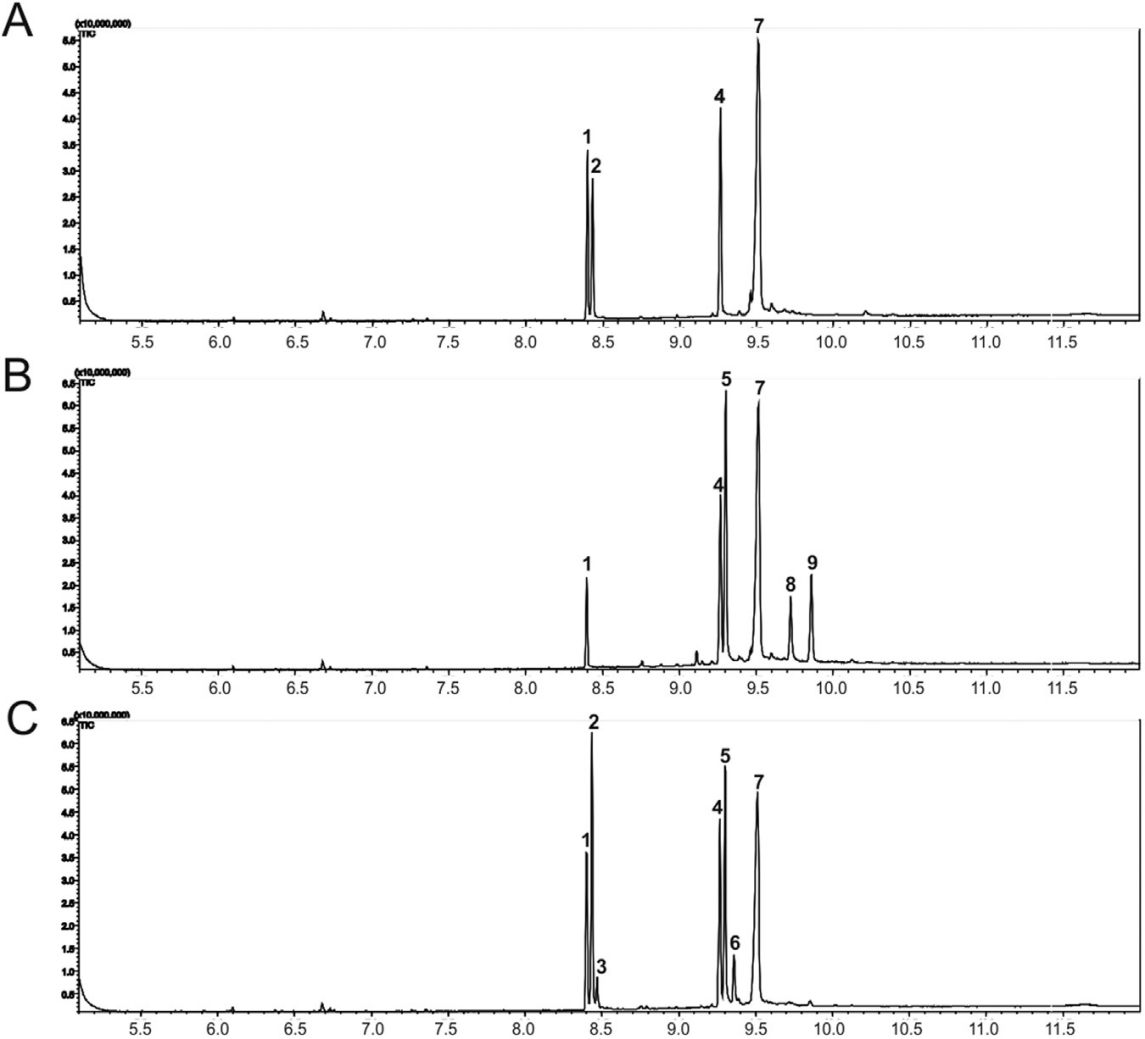
GC-MS total ion chromatograms of *P. pastoris* strains after 72 h of induction. A: strain P_PIS1_-*ERG1* SHC D377C B: strain P_PIS1_-*ERG1* TC C: strain P_PIS1_-*ERG1* TC D373C. Compounds detected in cell pellet: squalene (1), 3-deoxyachilleol (2), presumably bicyclic squalene derivative (3), cholesterol-TMS ether (4, internal standard), 8α-hydroxypolypoda-13,17,21-triene (5), (+)-ambrein (6), ergosterol-TMS ether (7), 14β-hydroxyonocera-8(26)-ene (8), onoceranoxide (9).

**Fig. S5:**
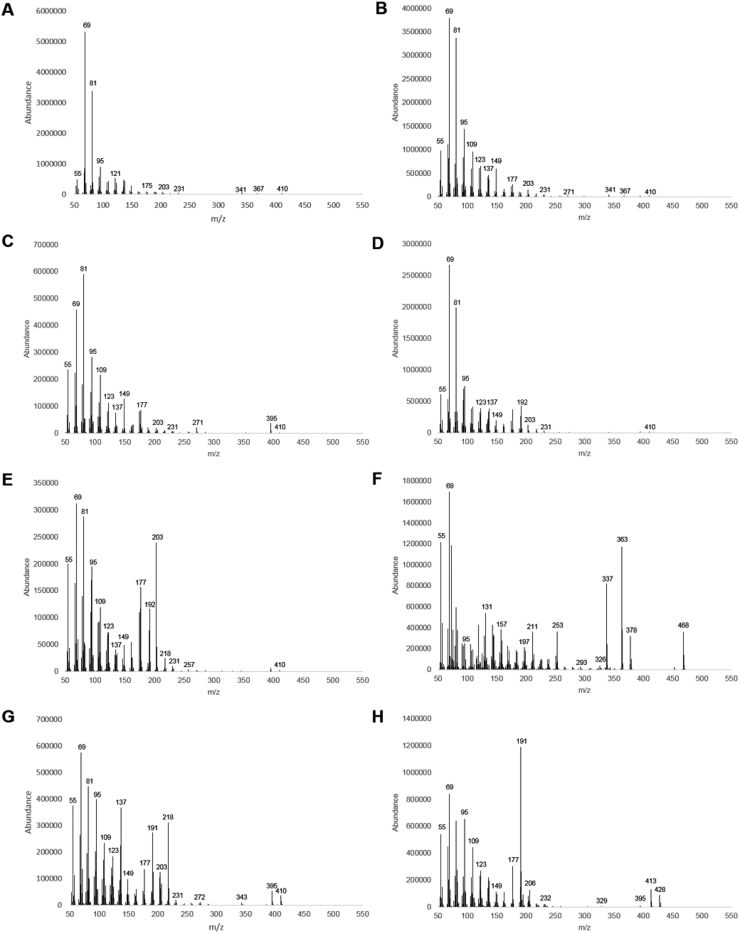
Mass spectra of the compounds shown in the chromatograms in Fig. 4. A: squalene, B: 3-deoxyachilleol, C: presumably bicyclic squalene derivative, D: 8α-hydroxypolypoda-13,17,21-triene, E: (+)-ambrein F: ergosterol-TMS ether G: 14β-hydroxyonocera-8(26)-ene, H: onoceranoxide.

**Fig. S6:**
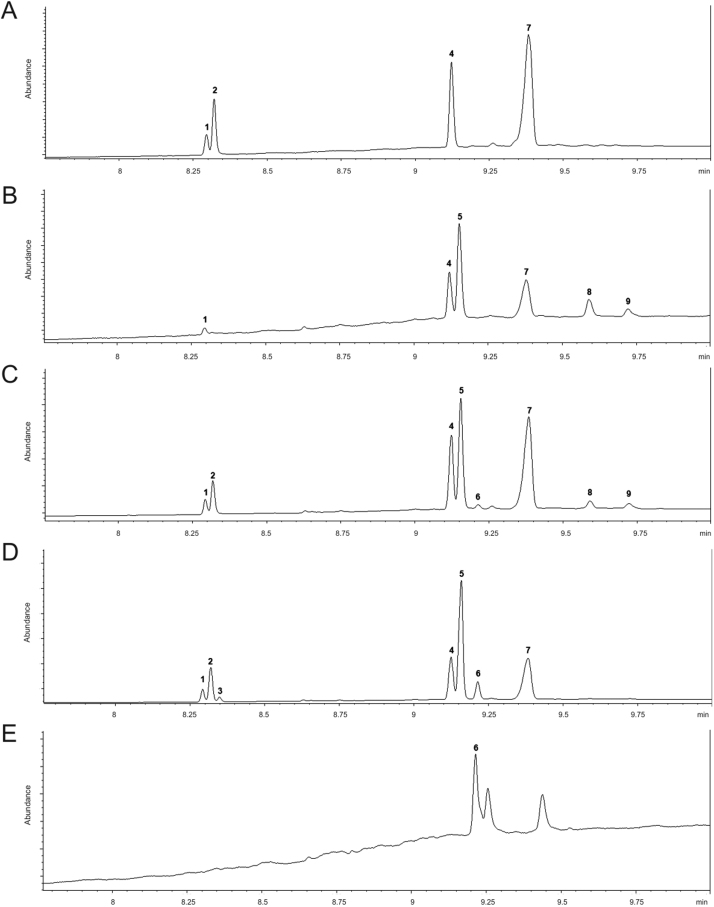
GC-FID chromatograms of P. pastoris extracts after 72 h of induction. A: strain PpPIS1-ERG1/SHC B: strain PpPIS1-ERG1/TC C: PpPIS1-ERG1/SHC/TC D: strain PpPIS1-ERG1/TC-D373C E: authentic ambergris sample. Compounds detected in cell extracts: squalene (1), 3-deoxyachilleol (2), presumably bicyclic squalene derivative (3), cholesterol-TMS ether (4, internal standard), 8α-hydroxypolypoda-13,17,21-triene (5), (+)-ambrein (6), ergosterol-TMS ether (7), 14β-hydroxyonocera-8(26)-ene (8), onoceranoxide (9).

After confirming expression of *Aa*SHC D377C and *Bme*TC, the next step was to establish analytical methods that allowed separation of all triterpenoids that would be produced. First analyses of *P. pastoris* strains expressing *Aa*SHC D377C with an established GC-MS method (Ueda et al., 2013) indicated that 3-deoxyachilleol was formed as the squalene peak showed a shift of its maximum. Our work confirmed the findings of Ueda et al. (2013) who stated that these two compounds cannot be separated easily due to their highly similar chemical structure. It should not go unnoticed that contradicting work was published recently (Ke et al., 2018). Consequently, a GC-MS method suitable to separate highly similar compounds such as squalene/3-deoxyachilleol and 8α-hydroxypolypoda-13,17,21-triene/(+)-ambrein was of utmost importance and therefore established (Fig. 4). Squalene and ergosterol were identified using authentic standards. To confirm 3-deoxyachilleol production, the compound was purified by RP-HPLC and was subsequently identified by NMR (see section 3.3.1 and supplemental information). (+)-Ambrein was identified by mass spectrometry (Fig. S5) and comparison to published results (Rowland and Sutton, 2017), and by using an authentic ambergris standard to confirm (+)-ambrein retention time in GC-FID (Fig. S6). Furthermore, Ueda et al., 2013, described that *Bme*TC produces 8α-hydroxypolypoda-13,17,21-triene, 14β-hydroxyonocera-8(26)-ene and onoceranoxide from squalene, which correspond - based on mass spectrometry (Fig. S5) – to peaks number 5, 8 and 9 in Fig. 4. A novel, as yet unidentified product was detected for the *Bme*TC D373C variant (Fig. 4C, peak 3). We suggest this compound to be a bicyclic derivative of squalene/3-deoxyachilleol based on mass spectroscopy and a strikingly consistent retention time shift observed for squalene (Fig. 4C, peak 1, no cyclic structure), 3-deoxyachilleol (Fig. 4C, peak 2, one cyclic structure) and the novel compound (Fig. 4C, peak 3, 2 cyclic structures proposed). Furthermore, the mass spectrum of this new product (Fig. S5) shows high similarity to *bis*-(6,11-cyclofarnesa-2,7(14)-diene), that is a squalene-derived, symmetric triterpenoid with six-carbon rings at each end of the molecule (Behrens et al., 2000), which corresponds to 3-deoxyachilleol being cyclized also at the other terminus.

Analysis of mass spectra (Fig. S4) revealed that when *N,O*-bis(trimethylsilyl)trifluoroacetamide was used for derivatization, ergosterol and the internal standard cholesterol were completely converted to corresponding trimethylsilyl (TMS) ethers while silylation of the tertiary alcohols 8α-hydroxypolypoda-13,17,21-triene, (+)-ambrein and 14β-hydroxyonocera-8(26)-ene was not successful corresponding well to the findings of Rowland and Sutton (2017). For routine analyses, a GC-FID method was developed (Fig. S6).

### 3.4 Identification of 3-deoxyachilleol by NMR

**Fig. S8:**
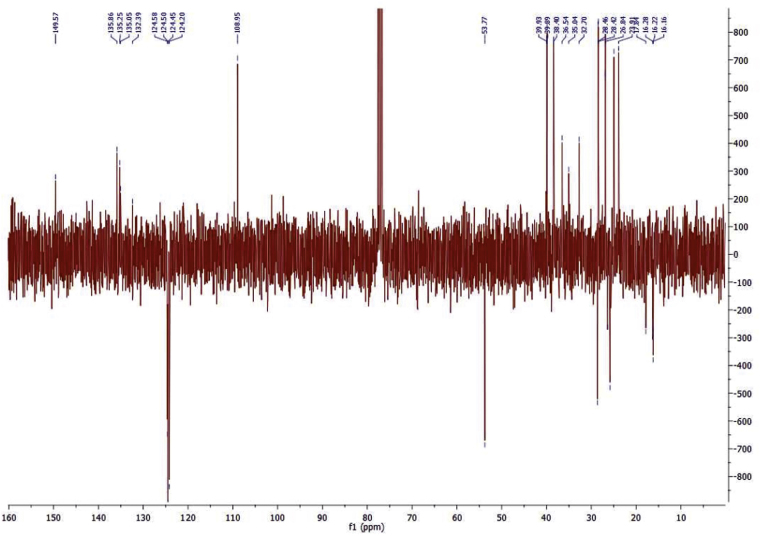
^13^C NMR spectrum (APT) of 3-deoxyachilleol in CDCl_3_.

^1^H- and ^13^C NMR data of purified 3-deoxyachilleol (Figs. S8 and S9): ^1^H NMR (300 MHz, CDCl_3_): δ = 5.12 (4H, m), 4.75 (1H, bs), 4.54 (1H, bs), 2.17–1.87 (14H, m), 1.82–1.70 (2H, m), 1.68 (3H, s), 1.60 (12H, s), 1.55 – 1.35 (5H, m), 1.30–1.10 (2H, m), 0.91 (3H, s), 0.83 (3H, s). ^13^C NMR (75 MHz, CDCl_3_, APT): δ = 149.57, 135.86, 135.25, 135.05, 132.39, 124.58, 124.50, 124.45, 124.20, 108.95, 53.77, 39.93, 39.89, 38.40, 36.54, 35.04, 32.70, 28.60, 28.46, 28.42, 26.94, 26.84, 26.37, 25.84, 24.94, 23.91, 17.84, 16.28, 16.22, 16.16.

The data matches the reported values from literature for 3-deoxyachilleol (Sato and Hoshino, 1999).

### 3.5 Positive impact of P_PIS1_-ERG1 on AaSHC D377C and BmeTC productivity

**Fig. 5:**
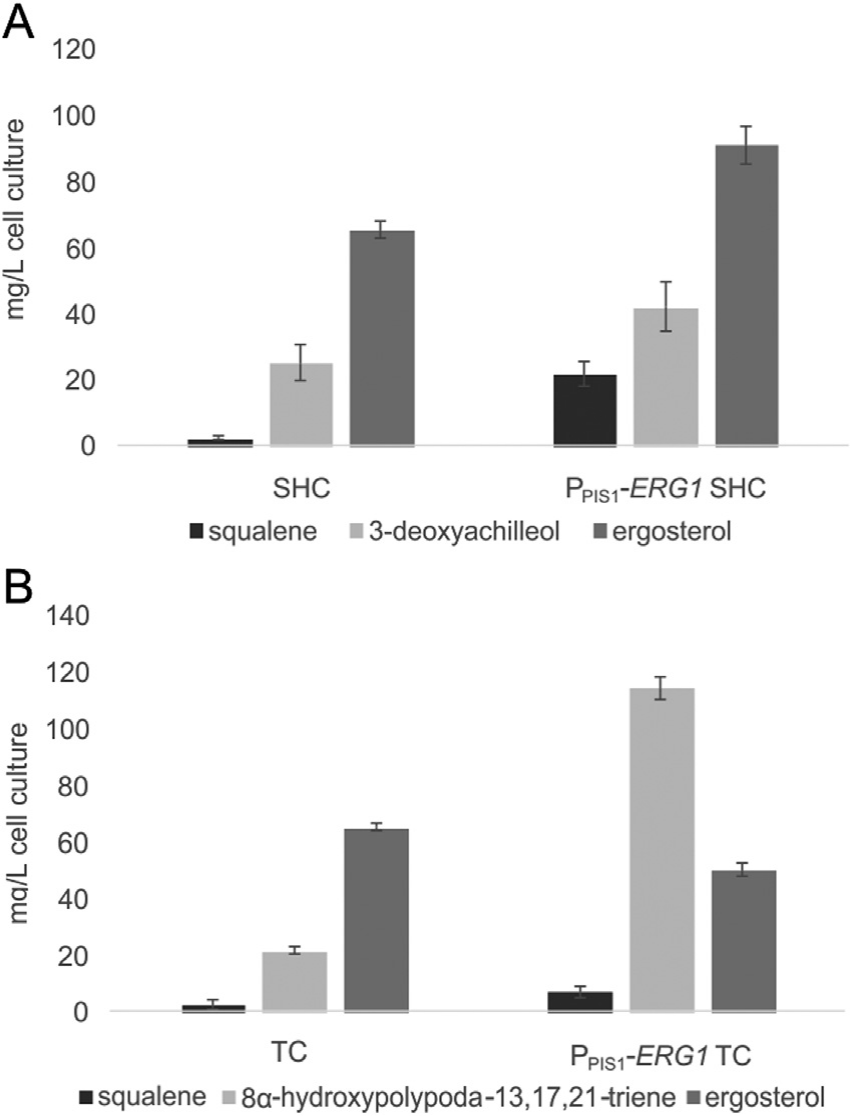
Triterpenoid levels in strains SHC (D377C) and P_PIS1_-*ERG1* SHC (D377C) (A) or TC and P_PIS1_-*ERG1* TC (B) after induction for 72 h (48 h of induction for *Bme*TC) in media supplemented with 0.1 µg mL^−1^ of terbinafine. Mean values and standard deviations of biological triplicates are given.

Considering the beneficial effect of terbinafine combined with exchanging the native *ERG1* promoter on squalene accumulation, *Aa*SHC D377C or *Bme*TC were expressed in this strain background. Triterpenoid and sterol levels were assessed after 72 h of induction in the presence of terbinafine. For *Aa*SHC D377C (Fig. 5A), the effect of P_PIS1_-*ERG1* on 3-deoxyachilleol production levels was visible but not as pronounced as might have been anticipated based on the results in Fig. 2. Substantial amounts of squalene were still present and ergosterol levels were slightly elevated, indicating that squalene was not consumed by *Aa*SHC D377C and was indeed converted to sterol. In contrast, for *Bme*TC based productivity, the P_PIS1_-*ERG1* background was highly beneficial, increasing 8α-hydroxypolypoda-13,17,21-triene yield by approximately four-fold. The efficient flux of squalene towards 8α-hydroxypolypoda-13,17,21-triene also resulted in decreased ergosterol levels. Collectively, this suggests limitations in the productivity of *Aa*SHC D377C, since Sato and Hoshino (1999) reported that *in vitro* the *Aa*SHC D377C variant exhibited a strongly decreased activity compared to the wild type enzyme.

### 3.6 Whole cell (+)-ambrein production with AaSHC -D377C and BmeTC

After establishing that both *Aa*SHC D377C and *Bme*TC exhibited their expected activity in *P. pastoris*, the enzymes were co-expressed in the same strain. To provide sufficient amounts of 3-deoxyachilleol as substrate for *Bme*TC and thereby improve the relative amounts of (+)-ambrein compared to 8α-hydroxypolypoda-13,17,21-triene, *Aa*SHC D377C expression was induced for 24 h before expressing it in parallel with *Bme*TC. GC-FID analysis of the cultures Table 2 revealed that the main products of strain P_PIS1_-*ERG1* SHC D377C TC were 3-deoxyachilleol and 8α-hydroxypolypoda-13,17,21-triene. However, a small amount of (+)-ambrein, approximately 2 mg L^−1^, could also be detected.

**Table 2:** Triterpenoid levels in strains P_PIS1_-*ERG1* SHC (D377C) TC and P_PIS1_-*ERG1* TC D373C after induction for 72 h in media supplemented with 0.1 µg mL^−1^ of terbinafine. Mean values and standard deviations of biological triplicates are given.

	**P**_**PIS1**_**-*****ERG1*****SHC (D377C) TC**	**P**_**PIS1**_**-*****ERG1*****TC-D373C**
**squalene [mg L**^**−1**^**]**	15.9 ± 4.6	20.1 ± 4.9
**3-deoxyachilleol [mg L**^**−1**^**]**	29.3 ± 7.4	63.1 ± 4.6
**8α-hydroxypolypoda-13,17,21-triene [mg L**^**−1**^**]**	68.2 ± 12.6	108.2 ± 7.6
**(+)-ambrein [mg L**^**−1**^**]**	1.9 ± 0.2	14.9 ± 2.1
**ergosterol [mg L**^**−1**^**]**	86.4 ± 7.6	58.9 ± 4.8

### 3.7 Engineering of BmeTC

The results for strain P_PIS1_-*ERG1* SHC D377C TC showed that, while (+)-ambrein was produced to some extent, there seemed to be limitations regarding the efficiency of the enzyme cascade. Distinct amounts of squalene and, remarkably, also 3-deoxyachilleol were found in the cells. Therefore, engineering of *Bme*TC was considered a promising approach to eventually improve the conversion of 3-deoxyachilleol. Although there was no *Bme*TC structure available and the sequence homology to the closest-related structure, strikingly *Aa*SHC, is only around 30%, certain amino acid stretches are conserved (Sato et al., 2011). Among these conserved regions we identified the DXDD motif, which has been previously shown to be of central importance for *Aa*SHC-catalyzed reactions. Specifically the D377C exchange led to a shift in the product spectrum from hopene to 3-deoxyachilleol (Sato and Hoshino, 1999). Therefore, this particular amino acid exchange was introduced forming the *Bme*TC D373C variant by site-directed mutagenesis. Analysis of triterpenoids of strain P_PIS1_-*ERG1* TC D373C (Fig. 4C and Table 2) surprisingly revealed that 3-deoxyachilleol was produced in significant amounts that were comparable to those of strain P_PIS1_-*ERG1* SHC D377C. Furthermore, also considerable 8α-hydroxypolypoda-13,17,21-triene levels were detected while 14β-hydroxyonocera-8(26)-ene and onoceranoxide were below detection limit. Remarkably, the strain expressing *Bme*TC D373C also produced clearly more (+)-ambrein, *i.e.* 15 mg L^−1^ in shake flask cultivation, than we achieved through co-expressing *Aa*SHC D377C and *Bme*TC.

The DXDD motif, which was not only found in squalene-hopene cyclases but also in class II diterpene cyclases (Prisic et al., 2007), is described to be essential in stabilizing the carbocation during conversion of squalene to hopene. When the last aspartic acid in the motif was exchanged for cysteine, the main product of *Aa*SHC became the monocyclic 3-deoxyachilleol instead of the pentacyclic hopene (Sato and Hoshino, 1999). We were highly surprised that by introducing this mutation into *Bme*TC, the enzyme produced 3-deoxyachilleol but still kept its original ability to generate the bicyclic 8α-hydroxypolypoda-13,17,21-triene. Moreover, it generated (+)-ambrein through its promiscuous activities. However, the tetra- and pentacyclic products 14β-hydroxyonocera-8(26)-ene and onoceranoxide were hardly detectable for the *Bme*TC D373C variant. This appears to correlate with the findings that the exchange of aspartic acid for cysteine at this position in *Aa*SHC hindered the formation of multicyclic products due of instability of the cation (Sato and Hoshino, 1999). This hypothesis is also supported by the fact that, in contrast to squalene-hopene cyclases, the related enzyme class of oxidosqualene cyclases contains an asparagine at the position corresponding to D376 in *Aa*SHC while the subsequent amino acid is a cysteine instead of asparagine. Thereby, oxidosqualene cyclases are not able to protonate a carbon-carbon double bond (Gao et al., 2012). Initial studies on the catalytic mechanism of *Bme*TC applying homology modelling and subsequent testing of active site variants have been published very recently (Tenkovskaia et al., 2017). It was shown that despite a high similarity of the active site architecture between *Aa*SHC and *Bme*TC, *Bme*TC exhibits a, so far, unique catalytic mechanism. Also, phylogenetic comparison of squalene-hopene cyclases sequences derived from a range of different bacteria with their homologues found in various *Bacillus* strains revealed that the second type forms an outgroup from all other bacterial SHCs (Bosak et al., 2008). Solving the *Bme*TC structure and additional mutational studies would clearly contribute to the detailed understanding of its reaction mechanism.

### 3.8 Cultivation of P_PIS1_-ERG1 TC D373C in bioreactor

**Fig. 6:**
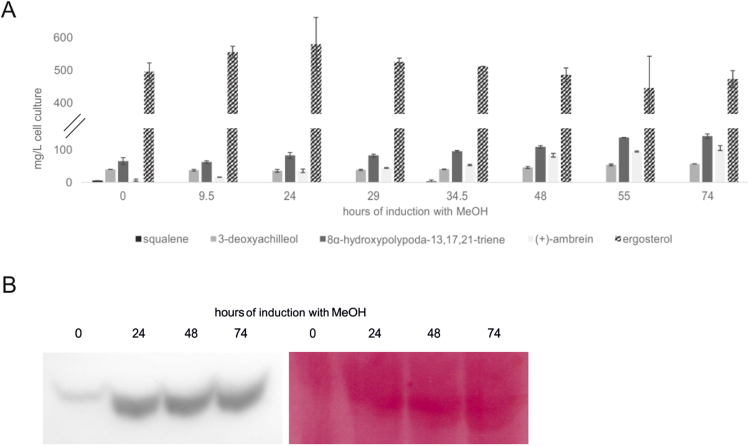
A: Triterpenoid levels in P_PIS1_-*ERG1* TC D373C cultivated in bioreactor. Samples were taken at different time points during methanol induction phase and culture volume corresponding to 10 mg of CDW was prepared for GC-FID analysis. The displayed data represent duplicate measurements. B: Immunoblot analysis of *Bme*TC D373C protein levels during MeOH induction phase and PonceauS stain of the PVDF membrane.

To evaluate the potential of our most advanced strain for industrial purposes, P_PIS1_-*ERG1* TC D373C was cultivated in bioreactor at 5 L scale. Pre-tests in shake flasks had shown that despite relatively high concentrations of zinc in the standard bioreactor cultivation medium for *P. pastoris,* BSM*,* no growth inhibition of the engineered P_PIS1_-*ERG1* strain background was detected. Samples of the bioreactor culture were taken at different time points during the methanol induction phase and triterpenoid levels were assessed (Fig. 6A). Already before induction with methanol was started, marked amounts of 3-deoxyachilleol, 8α-hydroxypolypoda-13,17,21-triene and (+)-ambrein were observed. This finding correlates with data from Nakagawa et al. (2004) describing that P_FLD1_ promoter showed between 20% and 35% of activity when glycerol was used as carbon source instead of methanol. Furthermore, immunoblot analysis (Fig. 6B) confirmed that small amounts of *Bme*TC D373C were expressed already before induction. Upon induction with methanol, the relative amount of (+)-ambrein compared to 3-deoxyachilleol and 8α-hydroxypolypoda-13,17,21-triene increased significantly, indicating that - in the absence of detectable amounts of squalene – enhanced levels of *Bme*TC D373C were essential to confer both reactions unto the terpenoid substrates and form (+)-ambrein. After 74 h of induction, cultivation in 5 L bioreactors resulted in an (+)-ambrein production level of 105 mg L^−1^. Immunoblot analysis showed that *Bme*TC D373C levels were stable throughout the induction phase.

## 4 Conclusion

In this study, we demonstrate for the first time the potential of *P. pastoris* for accumulating substantial amounts of squalene by targeting a central enzyme in sterol metabolism, Erg1p. We established a yeast whole-cell system for (+)-ambrein biosynthesis by heterologously expressing two terpene cyclases, *Aa*SHC D377C and *Bme*TC that had been previously applied for conversion of squalene to (+)-ambrein in a two-step *in vitro* approach with *E. coli* as host. Engineering of *Bme*TC generated an enzyme that could catalyze the whole reaction from squalene to (+)-ambrein far more efficiently (factor of 7) compared to the previous two-enzyme system, yielding 15 mg L^−1^ cell culture of (+)-ambrein in shake flasks. Upscaling to 5 L bioreactors resulted in over 100 mg L^−1^ of (+)-ambrein, underlining the potential of this engineered *P. pastoris* strain as triterpenoid production platform. Combining cell and enzyme engineering approaches, (+)-ambrein yields of our strain clearly exceed production levels of the only other whole-cell system, *E. coli*, that had been reported so far (Ke et al., 2018). Metabolic and enzyme engineering approaches as well as adjusting cultivation and process conditions are highly promising and will further enhance (+)-ambrein yield.

## Figures and Tables

**Fig. S7:**
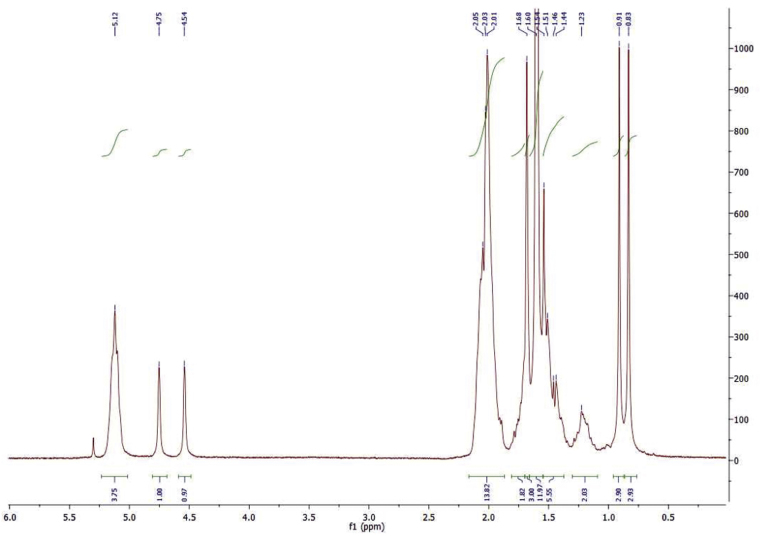
^1^H NMR spectrum of 3-deoxyachilleol in CDCl_3_.
