# Supplementary material for: Whole-cell (+)-ambrein production in the yeast Pichia pastoris
Source: Metab Eng Commun. 2018 Aug 16;7:e00077. doi: 10.1016/j.mec.2018.e00077 (PMC6127371; doi:10.1016/j.mec.2018.e00077)
Supplement: Supplementary file 1 — Supplementary material [file mmc1.docx]

**Supplemental information**

**Table S1 Oligonucleotide primers used in this study**

|  | Primer | Sequence in 5’ to 3’ direction |
| --- | --- | --- |
| 1 | **Fw_5'Erg1** | gcgccgctacagggcgcgtatttaaatatatgtacacaatgaatgacctaatcacc |
| 2 | **Rev_5'Erg1** | gatacgttccgttccacggtctttttaaatgaatgttatgg |
| 3 | **Fw_HIS4** | cataacattcatttaaaaagaccgtggaacggaacgtatcttagcat |
| 4 | **Rev_HIS4_ovPIS1** | gcaaaaacttttagcctcgttactcttgagataaatttcacgtttaaaatcag |
| 5 | **Fw_PIS1** | gaaatttatctcaagagtaacgaggctaaaagtttttgca |
| 6 | **Rev_PIS1** | ctctttcaggggacatcgtttcggactatctagagacaagtaaatttccatgttg |
| 7 | **Fw_Erg1_ovPIS** | ctagatagtccgaaacgatgtcccctgaaagagactacaatattatc |
| 8 | **Rev_3'Erg1_SwaI** | gcggagcctatggaaatttaaatggtcgctgtaggttacgg |
| 9 | **Fw_5'intERG1cassette** | caagctcccgggttattcaacg |
| 10 | **Rv(His4) AEM** | gtatcctggcttggcatc |
| 11 | **Fw(PIS1end) AEM** | tgtctctagatagtccgaaacg |
| 12 | **Rev_3'intERG1cassette** | gcttttgggctactttgtctaggtg |
| 13 | **Fw_Ecolipart_SwaI** | gtaacctacagcgaccatttaaatttccataggctccgcc |
| 14 | **Rev_Ecolipart_SwaI** | tgattaggtcattcattgtgtacatatatttaaatacgcgccctgtagcg |
| 15 | **Fw-pFLD1-SwaI** | ggatttaaatgcatgcaggaatctctggc |
| 16 | **Rev-pFLD1-EcoRI-SpeI** | ggactagtgaattctgtgaatatcaagaattgtatgaacaagc |
| 17 | **Fw_AaSHC_SpeIKzFLAG** | ggactagtcgaaacgatggattacaaggatgacgacgataaggcagagcaattggttgaagc |
| 18 | **Rev_AaSHC_NotI_new** | gttgcggccgcttaccttctctcgatagcctgc |
| 19 | **Fw_BmeTC_AscIKzmyc** | cttggcgcgcccgaaacgatggaacaaaaactcatctcagaagaggatctgattatcctgctgaaagaggtcc |
| 20 | **Rev_BmeTC_PacI** | ggttcttaattaattaggaagagtgcttcttagcgtag |
| 21 | **Fw_BmeTC_SpeI** | gaattcactagtcgaaacgatggaacaaaaactc |
| 22 | **Rev_BmeTC_NotI** | cttgagcggccgcttaggaagagtgcttcttagcgtagtg |
| 23 | **Fw_5'AOX1_XmaI** | ctgattttaaacgtgaaatttatctcacccgggaattcccgctttgactgcctgaaat |
| 24 | **Rev_5'AOX1_MCS** | aaatggcattctgacatcctcttgacctgcaggttaattaaggcgcgcctttcaataattagttgttttttgatcttctcaagttgtcg |
| 25 | **Fw_AOXTT_MCS** | atcaaaaaacaactaattattgaaaggcgcgccttaattaacctgcaggtcaagaggatgtcagaatgccatttg |
| 26 | **Rev_AOXTT_NotI** | gttcctattctctagaaagtataggaacttcgcggccgcgcacaaacgaaggtctcacttaatcttctgtac |
| 27 | **Fw_FLP_NotI** | cagaagattaagtgagaccttcgtttgtgcgcggccgcgaagttcctatactttctagagaataggaacttcagatctaacatccaaa |
| 28 | **Rev_FLP** | ccatacttccaatcatagatactcggaagttcctattctctagaaagtataggaacttcacatgtgagcaaaa |
| 29 | **Fw_3'AOX** | tttgctggccttttgctcacatgtgaagttcctatactttctagagaataggaacttccgagtatctatgattggaagtatgggaatgg |
| 30 | **Rev_3'AOX_XmaI** | gatttcaggcagtcaaagcgggaattcccgggtgagataaatttcacgtttaaaatcagcgtacct |
| 31 | **Fw_TC_D373C** | cccagacttggactgcacttccgctgctata |
| 32 | **Rev_TC_D373C** | tatagcagcggaagtgcagtccaagtctggg |


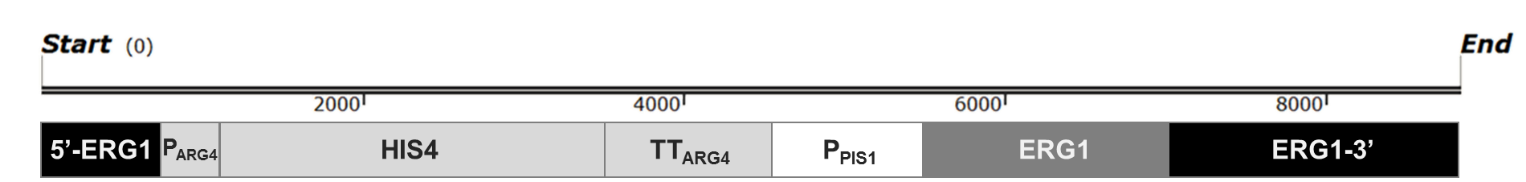


**Figure S1 Expression cassette to exchange the native promoter of ERG1 by the PIS1 promoter. Expression cassette contains homologous regions to the 5′- and 3′-flanking sequences of the ERG1 locus.**

**Figure S2 AaSHC and BmeTC sequences codon-optimize for expression in P. pastoris**


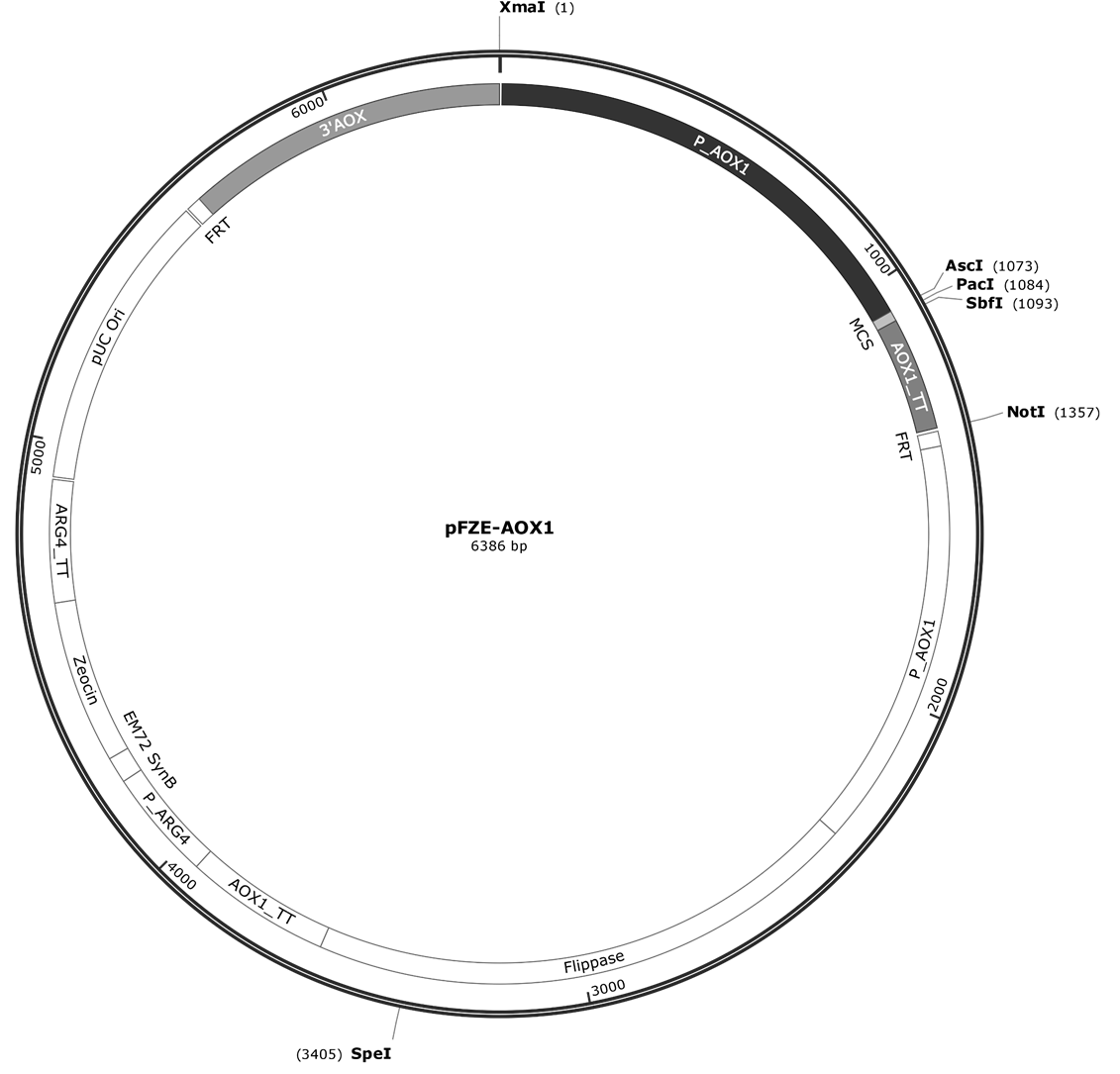


**Figure S3 Map of plasmid pFZE-AOX1.**


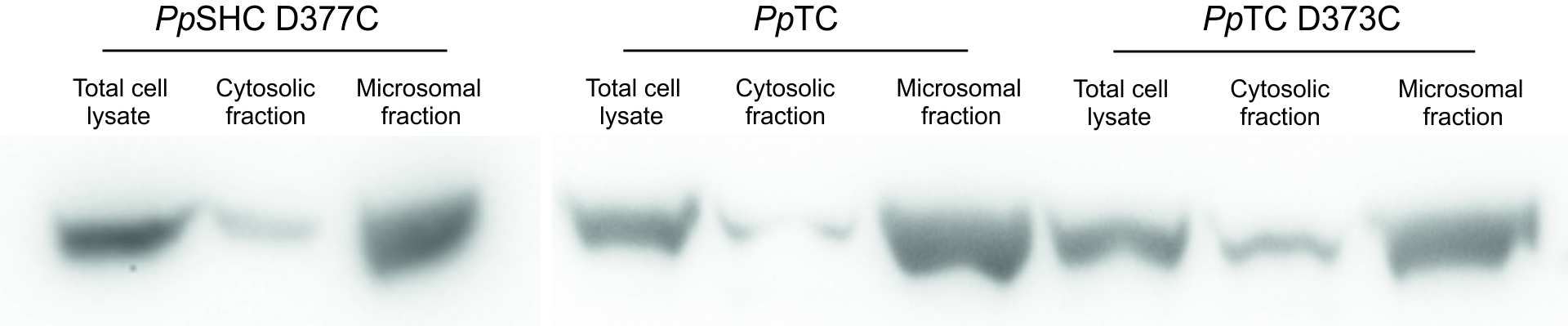


**Figure S4 Western Blot analysis using antibodies directed against FLAG-tag of FLAG-SHC D377C and myc-tag of myc-TC and myc-TC D373C in the respective P. pastoris (Pp) expression strains.**


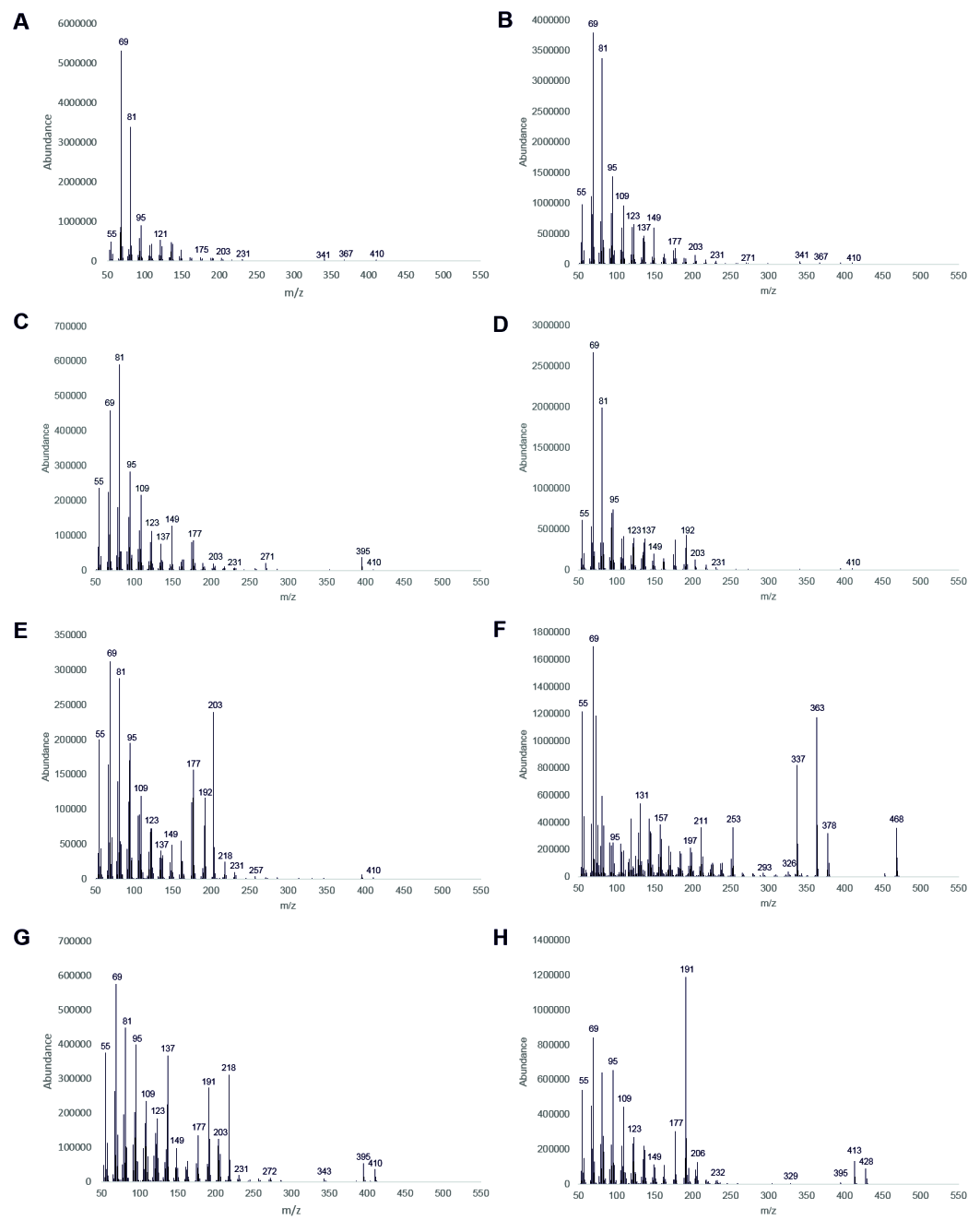


**Figure S5 Mass spectra of the compounds shown in the chromatograms in Figure 4. A: squalene, B: 3-deoxyachilleol, C:** **presumably bicyclic squalene derivative, D: 8α-hydroxypolypoda-13,17,21-triene, E: (+)-ambrein F: ergosterol-TMS ether G: 14β-hydroxyonocera-8(26)-ene, H: onoceranoxide.**


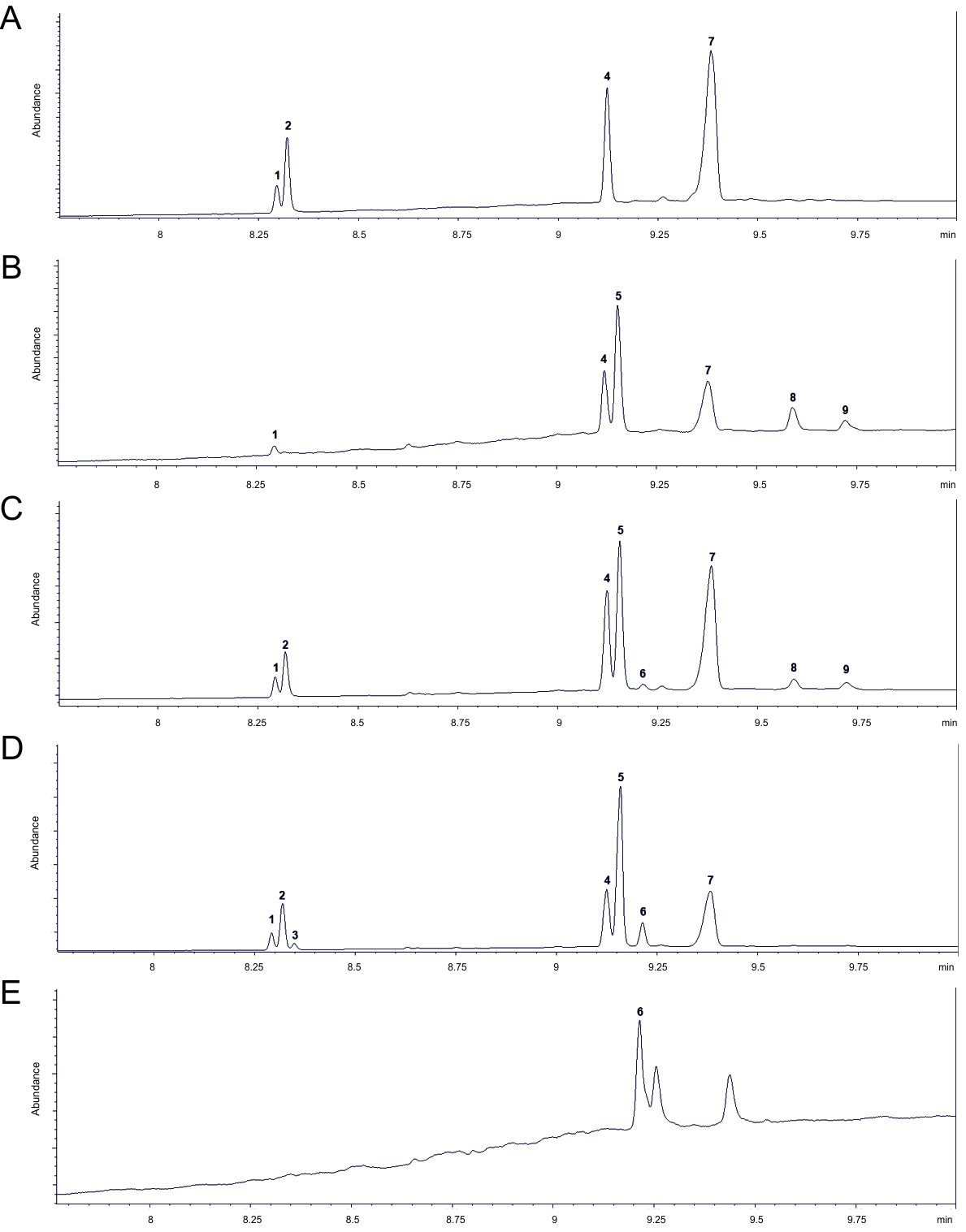


**Figure S6 GC-FID chromatograms of P. pastoris extracts after 72 h of induction. A: strain PpPIS1-ERG1/SHC B: strain PpPIS1-ERG1/TC C: PpPIS1-ERG1/SHC/TC D: strain PpPIS1-ERG1/TC-D373C E: authentic ambergris sample. Compounds detected in cell extracts: squalene (1), 3-deoxyachilleol (2), presumably bicyclic squalene derivative (3), cholesterol-TMS ether (4, internal standard),** **8α-hydroxypolypoda-13,17,21-triene (5), (+)-ambrein (6), ergosterol-TMS ether (7), 14β-hydroxyonocera-8(26)-ene (8), onoceranoxide (9).**

**Purification and identification of 3-deoxyachilleol**

RP-HPLC analysis: instrument: Agilent 1100 equipped with a DAD detector; column: EC 150/3 Nucleodur C18 Gravity 3 μm (Macherey-Nagel, Düren, Germany); analysis conditions: acetonitrile/methanol 95:5, isocratic, 0.70 mL·min^-1^ flow rate, 30°C, UV detection at 210 nm.


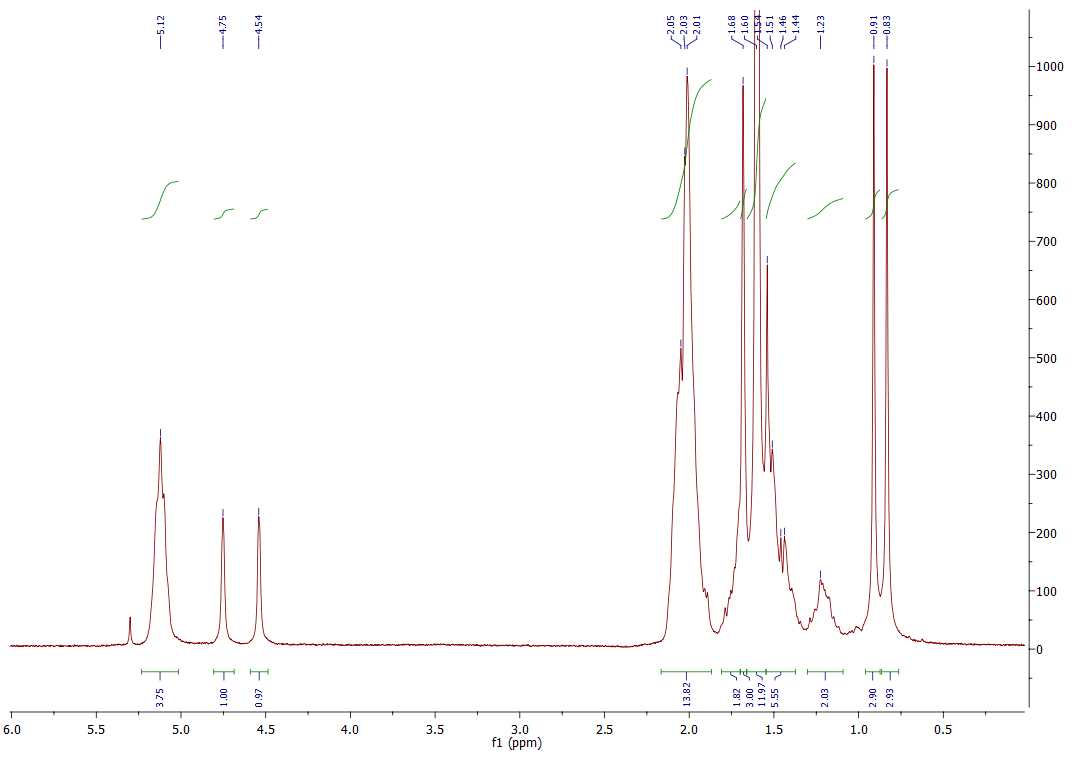


**Figure S7 ^1^H-NMR spectrum of 3-deoxyachilleol in CDCl_3_.**


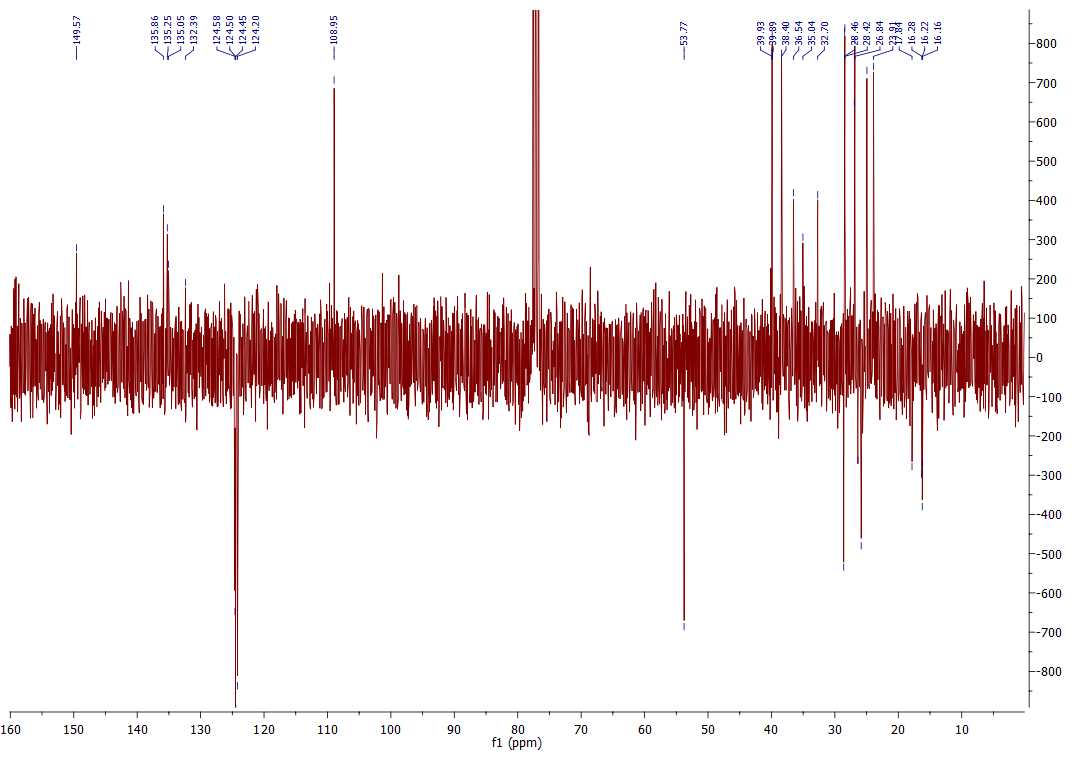


**Figure S8 ^13^C-NMR spectrum (APT) of 3-deoxyachilleol in CDCl_3_.**
